# Influence of starvation on walking behavior of *Bagrada hilaris* (Hemiptera: Pentatomidae)

**DOI:** 10.1371/journal.pone.0215446

**Published:** 2019-04-18

**Authors:** Ian M. Grettenberger, Shimat V. Joseph

**Affiliations:** 1 Department of Entomology and Nematology, University of California Davis, Davis, California, United States of America; 2 University of California Cooperative Extension, Salinas, California, United States of America; Montana State University Bozeman, UNITED STATES

## Abstract

*Bagrada hilaris* (Burmeister) (Hemiptera: Pentatomidae) is an invasive stink bug species that feeds on cruciferous plants and can cause substantial damage to crops. Little is known about the dispersal behavior of *B*. *hilaris*, but movement is important because of the way this pest moves from senescing weed hosts into crop fields. Perhaps, *B*. *hilaris* residing on declining weed hosts become starved, which alters their normal locomotor activity and initiates dispersal. We examined the influence of starvation on the locomotor behavior of multiple life stages of *B*. *hilaris* under laboratory and outdoor conditions. We starved nymph (2^nd^/3^rd^ and 4^th^/5^th^ instars) and adult (female and male) stages for 0, 24, and 48 h. We measured distance moved in the laboratory and then distance moved and turning ratio outdoors. In the laboratory, the younger nymphs moved shortest distances when starved for 24 h, whereas late-instar nymphs (4^th^-5^th^ instars) and adult *B*. *hilaris* that were starved moved farther than non-starved individuals. In the outdoor setting, environmental conditions, specifically surface temperature were important in determining how starvation affected distance moved. Starved insects were more responsive (moved farther) for a given change in temperature than non-starved insects. At lower temperatures, *B*. *hilaris* tended to move farther when non-starved and at higher temperatures, moved longer distances when starved, at least for certain stages. Increased starvation also led to more directional movement. Our results indicate that starvation influences aspects of movement for *B*. *hilaris* and that these effects can be influenced by temperature.

## Introduction

*Bagrada hilaris* (Burmeister) (Hemiptera: Pentatomidae), commonly known as bagrada bug or painted bug, is an invasive stink bug species that damages crops in California and the desert southwest [1,2]. The bagrada bug preferentially feeds on cruciferous crops (family: Brassicaceae) such as broccoli (*Brassica oleracea* var. *italica* Plenck), cauliflower (*B*. *oleracea* L. var. *botrytis*), turnip (*B*. *rapa* L. var. *rapa*), kale (*B*. *oleracea* L. *acephala*), bok choy (*B*. *rapa* L. var. *chinensis*), arugula (*Eruca sativa* Mill.) and mizuna (*B*. *rapa* L. *nipposinica*), and several other minor cruciferous crops [1,3,4]. This pest was first reported in North America in Los Angeles Co., California in 2008 [5] and then found in 2012 in the Central Coast region of California [2]. In California, *B*. *hilaris* could damage many different cruciferous crops and likely caused crop losses in the millions of US dollars when it first appeared in the region.

While *B*. *hilaris* will feed on all growth stages of cruciferous plants, its feeding on the young seedling stages is especially damaging. *B*. *hilaris* can kill seedlings, reduce plant stands, and severely stunt surviving plants when they feed in fields immediately following planting and when plants are young [1,3,4]. Damage on leaves, even when largely cosmetic, can render leafy cruciferous crops (e.g., arugula, mizuna, and kale) unmarketable. In broccoli and cauliflower, economic injury can occur when feeding by *B*. *hilaris* kills or damages the apical meristem of young seedlings. This damage causes plants to not produce a head or to produce multiple secondary shoots with undersized and unmarketable heads [5].

Several broad-spectrum insecticides, primarily pyrethroids and carbamates, have been effective for controlling *B*. *hilaris* [6,7]. Growers sometimes resort to multiple sprays of these insecticides at young stages of plant development to manage *B*. *hilaris* [7,8]. However, growers who choose to use no or minimal synthetic insecticides are at serious risk of crop damage due to lack of sustainable management tactics like biological control. Some growers of organic vegetables have even shifted to non-cruciferous crops late in the season to avoid losses to *B*. *hilaris* [1]. At times severe pest pressure and staggering economic losses in the Central Coast region in recent years have prompted a need to develop more refined integrated pest management programs for *B*. *hilaris*.

In the Central Coast, *B*. *hilaris* quickly emerged as a serious pest of cruciferous crops and has caused crop losses primarily during mid to late season, which spans from late July to late October [9]. Most of the persistent problems with *B*. *hilaris* have occurred in areas with higher temperatures, such as the southern areas of the Salinas Valley in Monterey County. Here, highs average 27–29 °C during July-October. These temporal and geographic patterns appear to result from how *B*. *hilaris* moves from non-crop hosts into crop fields in the surrounding landscape when the non-crop hosts senesce and disappear as a food source [1,10]. Populations of *B*. *hilaris* build on a number of cruciferous weed species, and large numbers of *B*. *hilaris* occur on these weed hosts mid to late season [1,11]. Cruciferous weeds such as shortpod mustard (*Hirschfeldia incana* (L.) Lagr.-Foss.) and perennial pepperweed (*Lepidium latifolium* L.) are common in non-crop and unmanaged areas in the Central Coast, including areas adjacent to crop fields (e.g., ditches, roadsides, and river bottoms; [12]). Weed management has been identified, and, in some cases, adopted as a key strategy to manage *B*. *hilaris* at the farm scale [8,11]. These large populations on weeds are the ones that seek out food resources (e.g., crops) and cause damage when weeds senesce and the insects are deprived of food. Large populations of *B*. *hilaris* and persistent feeding can further stress senescing weeds, accelerating senescence or even killing plants that might otherwise persist into the following year [10]. As the plants senesce and die, populations of *B*. *hilaris* will face food deprivation.

Food deprivation can be closely linked to dispersal for insects [13–18], but the dispersal behavior and movement ecology of *B*. *hilaris* is poorly understood. This information is critical for *B*. *hilaris* because of its propensity to rapidly arrive in fields, to disperse from non-crop areas, and to move across the landscape. Normal dispersal and locomotor behavior of insects is often altered by food deprivation [13,16]. Increased starvation is typically associated with increases in host searching behavior, directional movement, and increased orientation towards food [15,19–21]. Mixed populations of *B*. *hilaris* adults and nymphs face starvation when the weeds on which they are feeding senesce or are killed by the feeding. Changes in behavior with various levels of starvation could thus be important factors influencing how this pest moves from weeds to crop fields and between crop fields.

The objectives of this study were to determine 1) how starvation influences *B*. *hilaris* locomotor behavior in laboratory and outdoor settings and 2) how starvation influences orientation of *B*. *hilaris* toward food. We focused on multiple life stages of the pest throughout our study. In the laboratory, we examined total distance moved. In an outdoor setting, we examined both total distance moved and a component of movement behavior, the directionality of movement. This study also addressed the influence of environmental factors (such as surface temperature and wind speed) on walking behavior of food-deprived *B*. *hilaris* in an outdoor setting. This information could help better predict dispersal of *B*. *hilaris* and help develop management tactics, such as weed management or trap cropping, and refine the timing of insecticide applications to promote more precise integrated pest management programs.

## Materials and methods

### Insects

We collected adult *B*. *hilaris* from an organic broccoli field in San Lucas, CA in 2015 (36°01'01.3"N 120°55'00.5"W). We maintained a colony started with these insects in plastic containers of various sizes: 19.4 × 13.5 × 10.9 cm (L × W × H; 1.6 L), 24.4 × 18.2 × 15.8 cm (3 liter), and 34.0 × 20.1 × 12.4 cm (5 L) (Really Useful Boxes, Normanton, West Yorkshire, UK), and 19.4 × 16.5 × 11.4 cm (small size) and 33.3 × 19.4 × 11.4 cm (large size) (Sterilite, Townsend, MA). The lids of the rearing containers were modified to allow air circulation by cutting a large hole in the lid and covering the hole with mesh fabric (Cat. # 7250NSW, BioQuip, Rancho Dominguez, CA). A fresh piece of organic broccoli head (~20 g per container) was added every day as a food and water source. A layer of fluoropolymer resin, or Fluon (PTFE-30; Insect-a-Slip, BioQuip, Rancho Dominguez, CA), was painted along the smooth top inner surface of the container to prevent *B*. *hilaris* from escaping. The rearing containers were placed on a laboratory bench under incandescent bulbs that were always on. The heat generated by the incandescent lamps maintained air temperature at ~39 °C and relative humidity at ~20% in the containers. Relative humidity was maintained at a consistent level through the interplay of moisture from food, limiting and allowing airflow within the cages, and heat from the lamps. Nymphs and adults were kept in the same containers.

### Laboratory experiment

We first examined the walking behavior of *B*. *hilaris* and influence of starvation and stage on distance moved in the laboratory. Movement by *B*. *hilaris* often consists of walking on the soil surface and foliage, although adults will fly, especially when disturbed and at higher air temperatures (>32 °C; [22]). Four different “stages” of *B*. *hilaris* were tested in this assay: 2^nd^-3^rd^ instar nymphs (young nymphs), 4^th^-5^th^ instar nymphs (old nymphs), adult females, and adult males. Each stage was further divided into three starvation treatments. Insects were starved for 0, 24, or 48 h before starting the assay. *Bagrada hilaris* has five nymphal stages, and we determined stages using the description in Taylor et al. [23]. We transferred individuals (ca. 30) with an aspirator into new 1.6 L rearing containers for each starvation*stage treatment combination. These individuals were maintained under our standard rearing conditions. To start the assay, we transferred individual *B*. *hilaris* into arenas consisting of polystyrene Petri dishes (100 mm × 15 mm, Fisher Scientific, Hampton, NH). We tested 23–26 *B*. *hilaris* per stage*starvation treatment combination (except for 4^th^/5^th^ instar nymphs starved for 48 h, *n* = 16; S1 Table). The dishes were placed on the base of a document visualizer (Visualizer re-350, Canon USA, Inc., Melville, NY) which backlit the arena with fluorescent lights and increased contrast. Trials lasted 20 minutes, starting immediately after the insects were introduced. We captured videos of the arenas during this period with an Ethernet camera (acA1300-60gm, Basler, Inc., Exton, PA). Videos of two arenas were recorded concurrently for assays with young nymphs, whereas five arenas were recorded concurrently for old nymphs and adults. We recorded air temperature with four mini digital thermo-hygrometers (Avianweb, Mims, FL) at the four corners of the visualizer surface. All assays were conducted between 23.8 and 29 °C (averaged over four measurements). Total distance covered was calculated using Noldus EthoVision XT software (Version 11.5, Noldus Information Technologies, Wageningen, The Netherlands).

### Outdoor experiment

Using direct observation, we assessed the effect of starvation period and insect stage on outdoor walking behavior. Young nymphs (2^nd^-3^rd^ instar), old nymphs (4^th^-5^th^ instar), and adults (male and female) were used in the assays. Each stage treatment was divided into the three starvation treatments (0, 24, or 48 h of starvation). During the starvation period, insects were held under the previously described laboratory rearing conditions. Our methods for quantifying outdoor walking movement by *B*. *hilaris* were modified from those of Lee et al. [24]. The assay was conducted in an asphalt parking lot in Salinas, CA (36°39'04.2"N 121°37'20.3"W). The trials were conducted during the warmest part of the day (11:00 to 16:00) because *B*. *hilaris* are most active when it is warm. For the assay, we selected asphalt that was free of cracks because *B*. *hilaris* tend to follow cracks, which would have resulted in unnatural movement. We swept the pavement clean of debris before the start of each daily series of trials. The movement of individual *B*. *hilaris* and the influence of starvation period and stage on movement were assessed by marking their locations at regular intervals during a trial to measure total and net distance travelled. We chose to conduct the assay on asphalt rather than a location like a crop field with a surface of soil because the former made it possible to measure actual distance travelled via distance between two points. In a crop field, the textured soil surface could make this difficult and would introduce unaccounted for variability. Our primary aim was to compare between stage and starvation treatments and not measure actual field dispersal rates.

To start a trial, we marked the insect’s starting location with colored chalk on the asphalt and placed an individual *B*. *hilaris* on this spot. We marked the insect’s position every minute, and the trial lasted 15 min. The observer moved at least 5 m from the location of the *B*. *hilaris* after each observation to reduce any observer effect. The number of individuals tested varied for each stage*starvation combination (mean = 41, range 17–67; S1 Table). Total distance moved was calculated by summing the distances traveled between sequential marked points. Net distance moved was measured as the distance between start and final positions. A few adults took flight during the trial, particularly when the surface temperatures were >38 °C. These replicates were not included in the analysis.

We also measured environmental variables to account for environmental conditions in the analyses. Surface temperatures readings were recorded at three random locations in the experimental area using a laser temperature gun thermometer (infrared 10:1, model # 2267–20, Milwaukee Electric Tool Corporation, Brookfield, WI). Air temperatures were recorded using two digital thermometers (Model #00325, AcuRite, Lake Geneva, WI) placed on the pavement surface before the start of the assay, and values were averaged. Differences in temperature between soil and asphalt also be important, particularly if temperatures on asphalt were either much lower or higher than insects would typically experience on soil. We took temperature readings of bare soil with the laser temperature gun thermometer at a nearby production field during the trials and temperatures were generally comparable. Wind speed was recorded during the assays at a height of ~1.2 m using a handheld weather monitor (model # WM-4, Ambient Weather, Chandler, AZ). Wind speed was measured at the cardinal directions at the release location of each *B*. *hilaris* individual before and after the assay. All assays were conducted during cloud-free days. Light intensity was recorded using the “Light/Lux Meter” app (developed by Patrick Giudicelli for Apple iPhone 4s, Cupertino, CA). The average light intensity was 26,971.7 LUX (*N* = 234) across assays.

### Olfactometer assay

The influence of starvation on the movement of adult *B*. *hilaris* towards food was tested using a glass Y-tube olfactometer. The olfactometer was 1 cm in diameter (0.8 cm inner diameter), with a 7.6 cm long stem and two 5 cm long arms at a 78° angle from each other (Quark Glass, Vineland, NJ). The ends of both arms were connected to two 45 mL glass chambers. The openings on the far sides of the glass chambers were sealed using cotton plugs. Insects were given a choice between a food source (a piece of broccoli crown) and an empty chamber. We randomized which arm contained the food for each trial.

Adult *B*. *hilaris* (females and males) were starved for 0, 24, or 48 h as previously described. Individual insects were introduced into the base of the Y-tube olfactometer and given 10 min to make a choice. This length of time was chosen based on preliminary experiments in which we confirmed the majority of insects had responded and made a choice within 10 minutes. We sealed the base of the tube with a cotton plug after introducing the insect. A choice was recorded for an assay when the insect moved 2 cm or further into one of the arms of the Y and remained there for at least 5 s. The final choice and time taken to choose an odor source was recorded. We tested twenty females and twenty males for each starvation treatment. The olfactometer was placed in a 60.5 × 50 × 46.5 cm cardboard box lined with white paper to limit external visual stimuli. We thoroughly washed the olfactometer with 95% ethyl alcohol and soapy water between assays to remove any residual odor. All trials were conducted at room temperature (21.1–26.6 °C).

### Statistical analyses

All analyses were conducted in R [25]. For the walking assay in the laboratory, we analyzed distance moved with ANOVA using *lm* in the stats package. Values were square root-transformed to satisfy assumptions of the analysis. Individuals that did not move were excluded from the analysis. Fixed factors that were included were starvation treatment, stage, and the starvation*stage interaction. The interaction was significant, so we examined the simple effects of both starvation and stage using *testInteractions* from package phia with Holm-Bonferroni p-value adjustments for multiple tests and contrasts. Means were separated with *emmeans* from package emmeans and Holm-Bonferroni adjustment.

For the outdoor movement assay, total distance moved was analyzed with *lm* in the stats package. Total distance was square transformed. We first fit a model with three-way interactions between the two treatment factors and each environmental variable, as well as all two-way interactions. Interactions were omitted based on AIC comparisons. Only starvation*stage and starvation*surface temperature were included in the final model, along with the fixed factors starvation, stage, surface temperature, and wind speed. Surface and air temperatures measured for individual trials were significantly correlated (*r* = 0.70; *P* < 0.001), and surface temperature was likely more influential than air temperature on *B*. *hilaris* behavior; therefore, surface temperature rather than air temperature was used in the analysis. Wind speed was not recorded for a subset of replicates (female-0 h: 7 reps, male-0: 18, male-24: 11, male-48: 11; total = 47). Rather than simply omitting these replicates and only use replicates with wind speed measurements or not using wind speed in the analysis, we used a polynomial regression with cubic terms for air and surface temperatures to predict wind speed for these replicates (S1 Fig). This did not change the overall results of the analyses as compared to alternatives of omitting replicates with missing wind data, and including wind speed in the analysis improved the model. The starvation*stage interaction was examined by testing the simple effects of both starvation and stage using *testInteractions* from package phia with Holm-Bonferroni p-value adjustments. Means were separated with *emmeans* from package emmeans with Holm-Bonferroni adjustments and were tested at 25 °C, 32.8 °C (mean) and 40 °C for starvation. The starvation*surface temp interaction was visualized using *interact_plot* from package jtools. We compared slopes for different starvation treatments using pairwise contrasts with *glht* from the multcomp package, with Bonferroni p-value adjustment. We examined changes in amount of turning and meandering by calculating and analyzing the ratio between net and total distance moved (net/total; “turning ratio”). This can be viewed as a reasonably coarse assessment of movement directionality. Values close to 1 indicate minimal turning and directed movement in nearly a straight line, and low values close to 0 indicate high levels of turning. In some cases, net distance was slightly greater than total distance due to small variation in measurement accuracy, so these ratios values were set to 1. Ratios were logit transformed for the analysis (log_10_[x/{1-x}], with 0.01 subtracted from values because some equaled 1). We analyzed turning ratio with *lm* in the stats package. We first fit a model with three-way interactions between the two treatment factors and each environmental variable, as well as all two-way interactions. Interactions were omitted based on AIC comparisons and no interactions were included in the final model, nor were the environmental variables. The final model contained fixed factors for starvation and stage. Means were separated with *emmeans* from package emmeans with Holm-Bonferroni adjustments.

## Results

### Laboratory experiment

**Fig 1:**
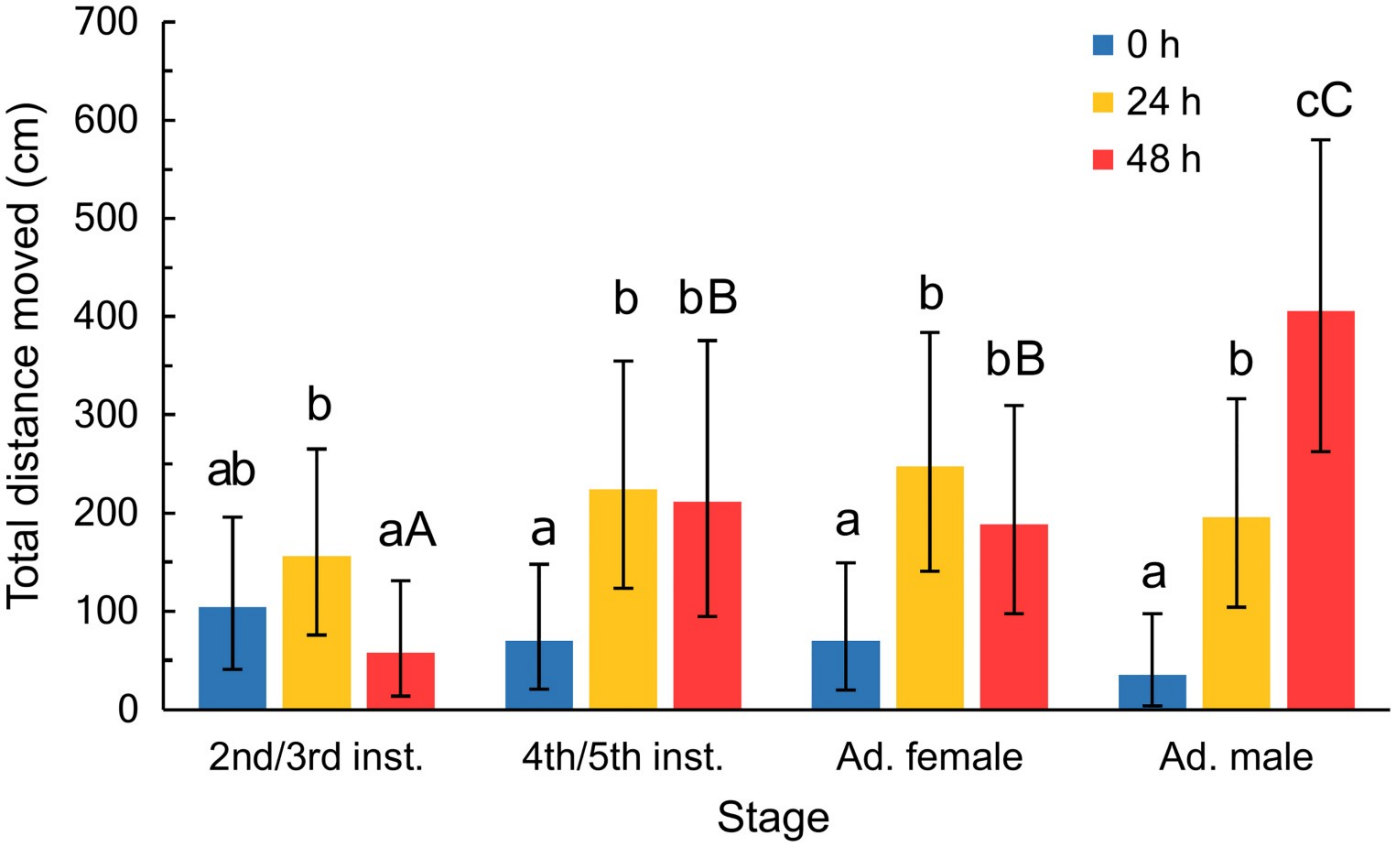
Total distance moved (cm) by different stages of bagrada bug that were starved for various time periods in the laboratory experiment. Insects were starved for 0, 24, or 48 h. Values were square-root transformed for the analysis. Means are back-transformed least-squares means along with their 95% confidence intervals. Within insect stages, starvation treatments that do not share a lowercase letter are significantly different based on posthoc pairwise comparisons. Uppercase letters indicate significant differences from posthoc pairwise comparisons between insect stages with only the 48 h starvation treatment. For pairwise comparisons, we used a Holm-Bonferroni p-value adjustment.

In total, we evaluated the walking behavior in the laboratory of 293 individual *B*. *hilaris*. The interaction between stage and starvation treatment was significant for distance moved (*F*_6, 281_ = 6.98; *P* < 0.001; Table 1, Fig 1). The simple effect of starvation treatment was significant within each stage (2^nd^/3^rd^ nymphs: *F*_2, 281_ = 3.39, *P* = 0.035; 4^th^/5^th^ nymphs: *F*_2, 281_ = 7.33, *P* = 0.002; females: *F*_2, 281_ = 8.16, *P* = 0.001; males: *F*_2, 281_ = 27.17; *P* < 0.001). For the 2^nd^/3^rd^ instar nymphs, insects starved for 24 h moved the most (2.1× more than those starved 48 h), with non-starved individuals moving an intermediate distance (Fig 1). Fourth/fifth instar nymphs starved for 24 h moved 2.6× farther than non-starved individuals. Fourth/fifth instar nymphs starved for 48 h moved 2.5× farther. The pattern of distance moved for females was very similar to that for 4^th^/5^th^ instar nymphs. Insects starved for 24 h moved 3.4× farther than non-starved insects and ones starved for 48 h moved 2.9× farther. For males, higher levels of starvation led to greater distances moved; compared to non-starved males, males starved for 24 h moved 4.7× farther and males starved for 48 h moved 9.8× farther. The simple effect analysis for starvation treatment indicated that stages moved different distances when they were starved for 48 h (*F*_3, 281_ = 14.41, *P* < 0.001), but there were no difference among stages when the insects were starved for 24 h (*F*_3, 281_ = 1.20, *P* = 0.35) or not starved at all (*F*_3, 281_ = 1.69, *P* = 0.34; Fig 1).

**Table 1:** Effect of starvation and stage on total distance moved in the laboratory experiment.

Factor	Sum of squares	*df*	*F*	*P*
Starvation	301	2	3.39	0.034
Stage	224	3	1.69	0.17
Starvation*stage	1855	6	6.98	< 0.001
Residuals	12442	281		

### Outdoor experiment

**Fig 2:**
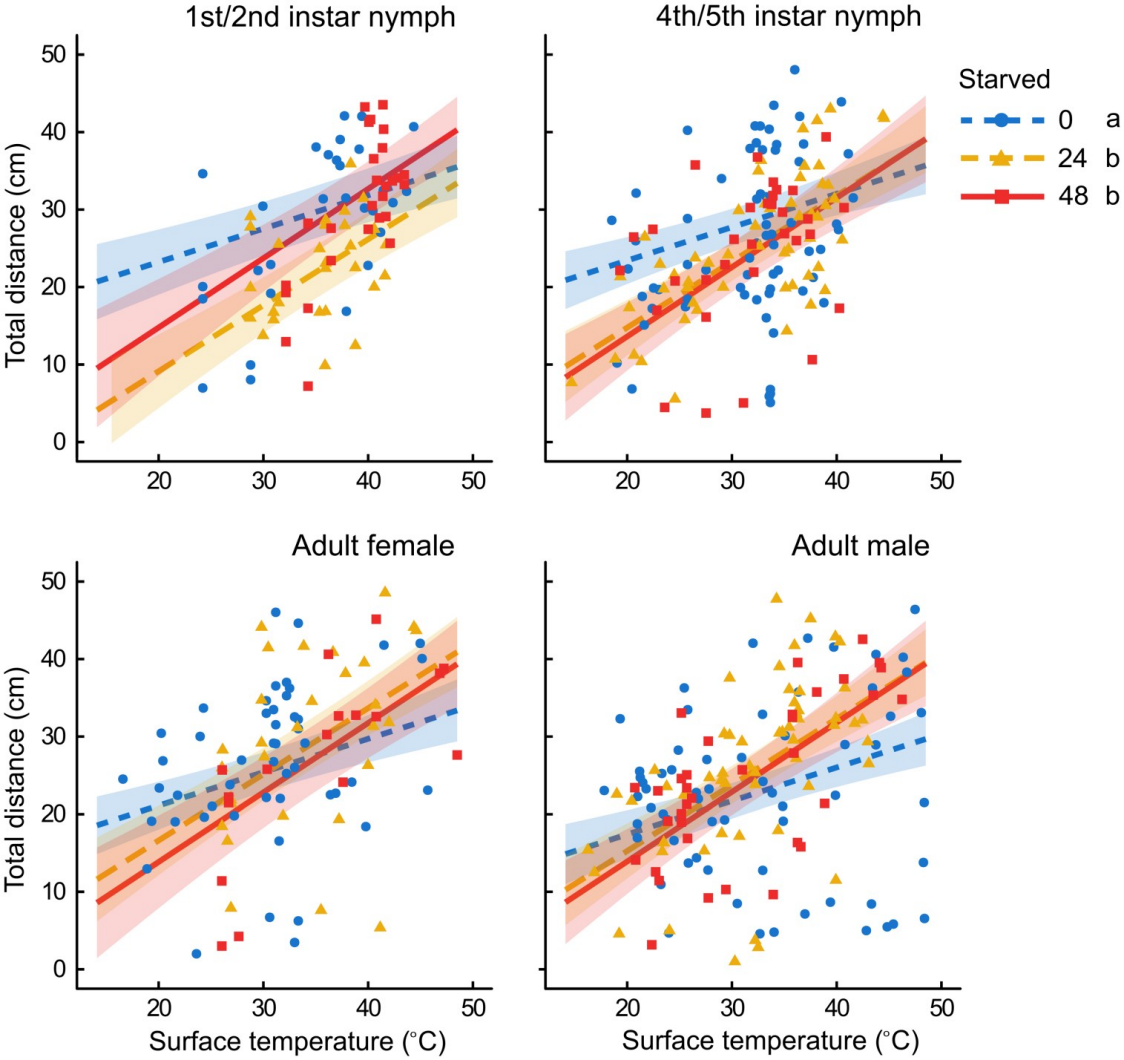
Total distance moved (cm) and surface temperature for various *B*. *hilaris* stages that were starved for different periods in the outdoor experiment. Tested stages were 2^nd^/3^rd^ instar nymphs, 4^th^/5^th^ instars, adult females, and adult males and they were starved for 0, 24, or 48 h. Lines represent relationships between total distance and surface temperature for each starvation treatment within each insect stage. Shaded regions are 95% confidence intervals. Values for total distance were square-root transformed for the analysis and these values are presented in the figure.

For total distance moved, both environmental variables (surface temperature and wind speed) were significant predictors of total distance moved and key factors in the model (Table 2). Wind speed had a reasonably weak but significant negative effect on total distance moved (wind speed mean±1 SD: 1.81±1.61; estimate = -1.31 [with distance square root transformed]). The effect of starvation treatment depended on surface temperature (significant starvation*surface temperature interaction; Table 2, Fig 2). Slopes differed between starvation treatments, with the steepest slopes (i.e., greatest increase in distance moved with increased temperature) for insects starved 24 or 48 h, which were significantly greater than for insects starved for 0 h (24 vs 0 h: *t* = 3.04, *P* = 0.007; 48 vs 0 h: *t* = 2.90, *P* = 0.011). The slopes for insects starved for 24 or 48 h were not significantly different from each other (*t* = 0.25, *P* = 1.00). The interaction between stage and starvation treatments was significant (Table 2). The effect of stage depended on starvation treatment, with an effect of stage for insects starved 0 h (F_3,476_ = 5.03, *P* = 0.006) and 24 h (F_3,476_ = 3.61, *P* = 0.027), but not 48 h (F_3,476_ = 0.10, *P* = 0.96; Fig 2). For 0 h of starvation, males traveled the least distance, with females intermediate, and both ages of nymphs the most. For 24 h, 2^nd^/3^rd^ instar nymphs traveled the least distance, with all other stages traveling equivalent distances. While there was no interaction between stage, starvation, and surface temp, because the relationship between total distance and surface temperature varied by starvation treatment, how starvation affected distance moved varied by stage and also at which surface temperature is chosen (Fig 2). Finally, males starved for different time periods did not travel varying distances at low temperatures, but as temperatures increased, insects starved for 24 or 48 h began to move farther than non-starved individuals. Testing for the effect of starvation at lower temperatures (25 °C) across life stages showed that insects that were not starved moved significantly farther than those starved for 24 or 48 h (0 h: 333 cm, 24 h: 345 cm, 48 h: 551 cm). At the mean temperature (32.8 °C) there were no significant differences between starvation treatments, although there was a trend towards starved insects moving shorter distances (0 h: 721 cm, 24 h: 620 cm, 48 h: 653 cm). At higher temperatures (40 °C), starvation did not significantly affect distance moved, although the trend was for distance moved increasing with starvation (0 h: 897 cm, 24 h: 963 cm, 48 h: 1023 cm).

**Table 2:** Effect of starvation, stage, and environmental factors on total distance moved in the outdoor experiment.

Factor	Sum of squares	*df*	*F*	*P*
Starvation	1578	2	9.52	< 0.001
Stage	1250	3	5.03	0.002
Surface temperature	2126	1	25.65	< 0.001
Wind speed	1465	1	17.68	< 0.001
Starvation*stage	1724	6	3.47	0.002
Starvation*surface temperature	1105	2	6.67	0.001
Residuals	39444	476		

**Fig 3:**
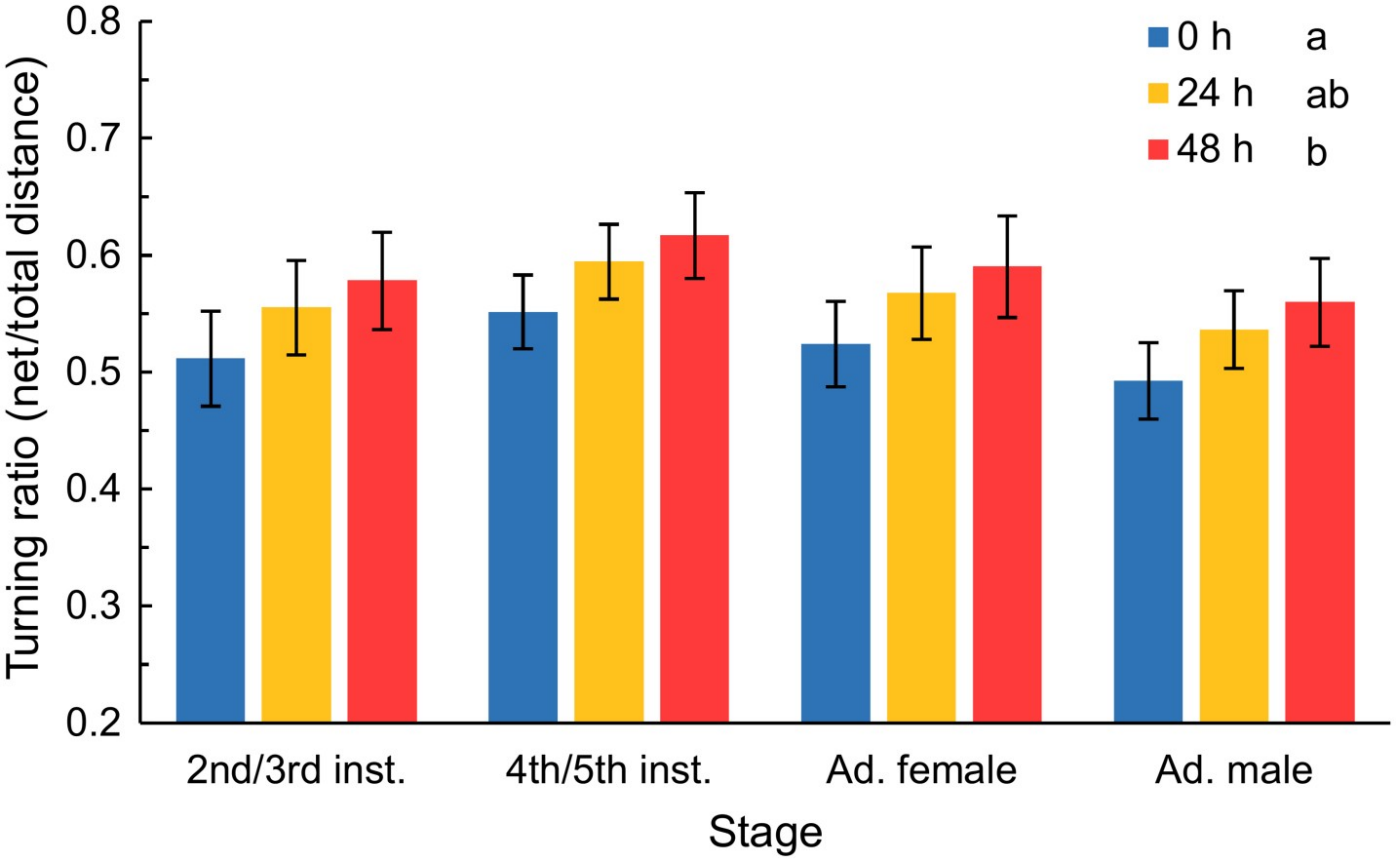
Turning ratio (net distance/total distance) for different stages of bagrada bug that were starved for different periods in the outdoor experiment. Insects were starved for 0, 24, or 48 h. Starvation significant influenced turning ratio across stages. Values were logit transformed for the analysis. Starvation treatments not sharing lowercase letters are significantly different based on posthoc comparisons. Means are back-transformed least-squares means with their 95% confidence intervals.

Both starvation and stage affected turning ratio, but there was no significant interaction between these factors (Table 3). The effect of starvation and treatment on turning ratio did not depend on surface temperature or wind speed and these variables were not even useful factors to include in the model for turning ratio. Increased levels of starvation led to more directional movement (Fig 3). Insects that were not starved turned the most, with net distance moved 52% of total distance. Insects starved for 24 h turned significantly less than those starved for 0 h, with net distance moved 56% of total distance. Insects starved for 48 h turned the least (statistically different than insects starved for 0 h but not 24 h), with net distance moved 59% of total distance. Across starvation treatments, male bugs turned the most (net distance 53% of total), which was significantly different than the turning ratio of 4^th^/5^th^ instars, which turned the least (net distance 59% of total). Adult females and 4^th^/5^th^ instars were intermediate between the other stages for turning ratio and not significantly different from each other or the other stages (net distance 56% and 55% of total distance, respectively).

**Table 3:** Effect of starvation and stage on turning ratio in the outdoor experiment.

Factor	Sum of squares	*df*	*F*	*P*
Starvation	5.5	2	6.55	0.002
Stage	4.1	3	3.26	0.022
Residuals	184.4	443		

Environmental factors did not account for variation in turning ratio. There was no interaction between starvation and stage.

### Olfactometer assay

**Fig 4:**
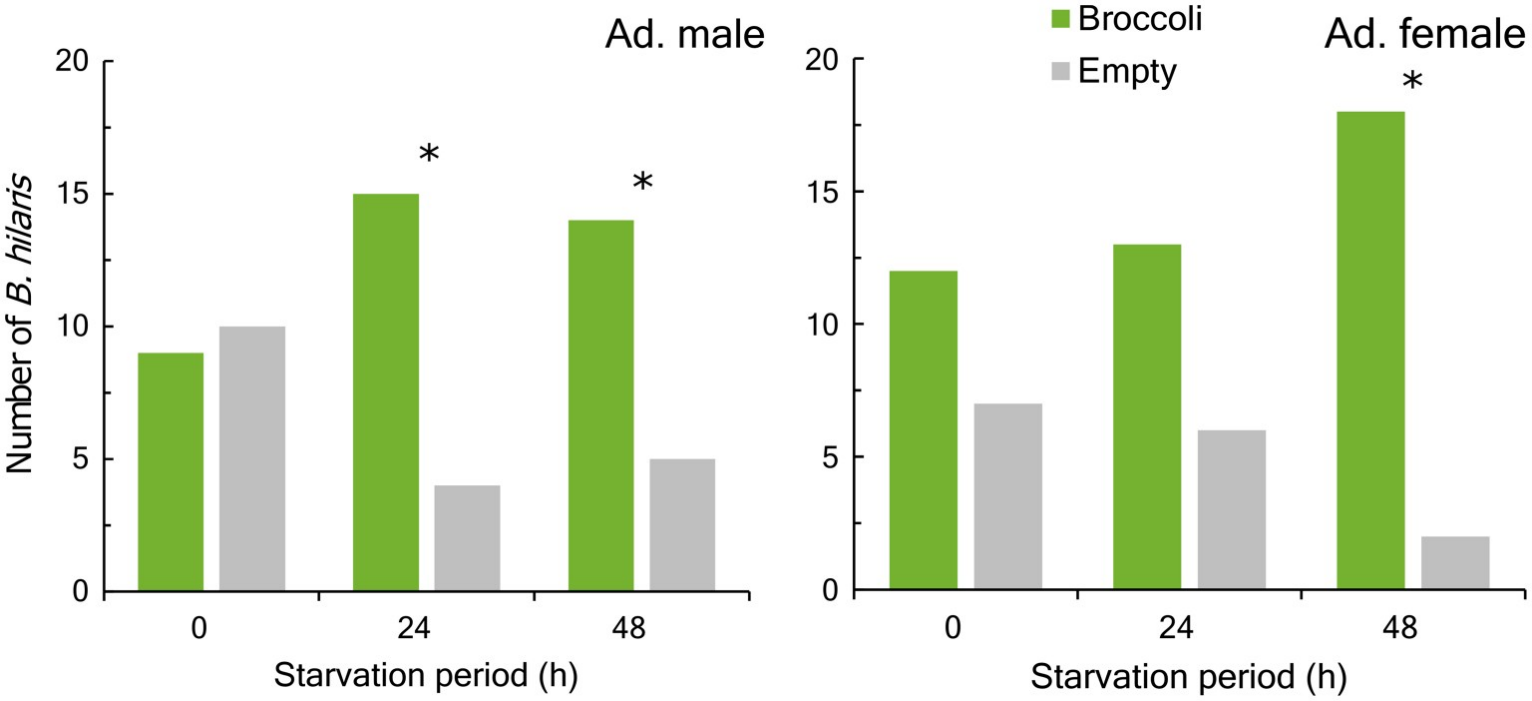
Effect of starvation period on orientation towards food in a two-arm olfactometer assay. Starvation periods were 0, 24, or 48 h and tested stages were adult male and female *B*. *hilaris*. Arms contained broccoli or were empty. Significant effects at α = 0.05 are indicated with an asterisk.

When both chambers were empty, *B*. *hilaris* showed no preference for either arm of the olfactometer (female: χ^2^ = 2.00, *P* = 0.18, *n* = 18; male: χ^2^ = 1.32, *P* = 0.25, *n* = 19). Female *B*. *hilaris* that were non-starved or starved for 24 h had no preference for arms that had broccoli or were empty (0 h: χ^2^ = 1.32, *P* = 0.25, *n* = 19; 24 h: χ^2^ = 2.57, *P* = 0.11, *n* = 19; Fig 4). After 48 h of starvation, significantly more female *B*. *hilaris* chose arms with broccoli than empty ones (χ^2^ = 12.80, *P* < 0.001, *n* = 20). Male *B*. *hilaris* showed no preference when starved for 0 h (χ^2^ = 0.05, *P* = 0.82, *n* = 19; Fig 4). Significantly more male *B*. *hilaris* moved to the arm with broccoli than the empty arm when starved for 24 (χ^2^ = 6.36, *P* = 0.012, *n* = 19) and 48 h (χ^2^ = 4.26, *P* = 0.039, N = 19).

## Discussion

Many polyphagous stink bug species feed primarily on plant fruiting structures and throughout the growing season search for fruits at a suitable developmental stage. To do so, they disperse among crop and non-crop hosts that differ in phenology [26,27]. The crucifer specialist *B*. *hilaris* differs in that it disperses within the landscape, at least in its introduced range in the U.S., based largely on the availability of any green tissue, leaves, stems, or fruiting structures, of its cruciferous hosts, which include weed and crop hosts [1,8]. Populations of *B*. *hilaris* appear to build and remain on weed hosts until the plants senesce, at which point they disperse in search of other hosts. A similar scenario can occur if *B*. *hilaris* populations develop on cruciferous crops that are then harvested or destroyed. Because hosts can rapidly disappear during the warmest part of the season when *B*. *hilaris* activity is the highest, starvation is likely one of the most important factors that influence movement and dispersal at this key period during the year. Our results demonstrate that starvation of *B*. *hilaris* can influence their dispersal behavior and orientation towards food. Our results indicate that life stages vary at times in their dispersal capacity and how they are affected by starvation. Environmental factors can also affect the influence of starvation on walking by *B*. *hilaris*. The change in locomotor behavior of *B*. *hilaris* with starvation provides insights into their dispersal behavior when feeding on senescing weed hosts.

Our results in a laboratory setting show that starvation tended to increase distance walked by *B*. *hilaris* (Fig 1). However, how the level of starvation affected movement varied with insect stage. Broadly speaking, food-deprived older nymphs (4^th^/5^th^ instars) and adults walked approximately two-fold further than non-starved individuals. For older nymphs and females, the distance walked was the same whether they were starved for 24 or 48 h. However, males moved increasing distances with greater levels of starvation. This effect of starvation on *B*. *hilaris* was broadly consistent with how other insect species have responded to starvation, which is a tendency to move more when starved [15,19–21]. Increased locomotor behavior is associated with a greater probability of finding a suitable food source [19]. In contrast, young nymphs (2^nd^/3^rd^ instar) in this assay moved the least when starved for 48 h, which was statistically different than when they were starved for 24 h. There was a non-statistical increase in movement with 24 h of starvation (vs. no starvation), which, coupled with the drop off in movement with 48 h of starvation, suggests that these earlier instars display a “hump-shaped” effect of starvation on movement [13]. Here, starvation initially increases movement, but there is subsequently a decrease in movement with longer periods of starvation. In this case, it is possibly because the small nymphs did not have sufficient energy reserves to withstand 48 h of starvation and still walk as far as they did when not starved. The larger nymphs and adults likely had sufficient energy reserves. Under these laboratory conditions, we also saw that the different life stages travelled different distances when they were starved for 48 h, but travelled equivalent distances when starved for 24 h or not starved. These results highlight how starvation can influence life stages differently, likely reflecting different patterns of dispersal or differences in physiology.

In an outdoor setting, we allowed the insects to move more naturally and tested how insect stage and starvation affected locomotor behavior under more variable environmental conditions. We were able to account for some of these variables in our model and attempted to control others (e.g., sunlight). Under these outdoor conditions, the influence of starvation on distance walked by *B hilaris*, and how the effect of starvation differed among stages, was driven by how environmental conditions affected walking by the insects. This did not result from wind, whose effect was straightforward (no significant interactions with treatments; Table 2), with increases in wind speed decreasing total distance moved. An important caveat is that we tested for an effect of wind speed, but not direction. It is possible that wind direction may have worked in combination with wind speed to influence movement if air was passing through patches of cruciferous weeds or across cruciferous crop fields. However, we did not account for this by mapping the surrounding landscape and relating this information to wind direction, although potential plant influences were not immediately adjacent to the testing area.

In contrast to wind speed, the influence of surface temperature on distance walked was more complex and varied by starvation treatment (Table 2, Fig 2). Higher temperatures led to greater distances moved. While this is not surprising for an ectotherm, and a species found in regions with high temperatures at that, it is notable that the nature of the relationship differed by starvation treatment. For all tested stages, insects that were starved were more responsive to increases in temperature than those that were not starved. The interactive effects of temperature and starvation on behavior in arthropods are generally unexplored [13]. In our study, starvation likely produced physiological changes in starved insects. This made them move further for a given increase in temperature because their energy reserves were more limited and finding a host was more imperative. It is notable that this effect was consistent across life stages. Across life stages, non-starved individuals moved the farthest at low temperatures, with a trend towards farther distances travelled with increasing starvation periods at high temperatures. The three-way interaction between stage, starvation, and surface temperature was not significant. However, coupled with the independent effect of stage, the significant starvation*surface temperature interaction suggests that for each life stage, the effect of starvation on distance moved may still depend on the chosen surface temperature (Fig 2). At lower temperatures, 2^nd^/3^rd^ instar nymphs moved shorter distances when they were starved than fed, although they moved similar distances at higher temperatures. They consistently moved further when starved for 48 h than for 24 h. The pattern for 4^th^/5^th^ instars was generally similar, although the effect of the two starvation treatments was identical. Female insects didn’t display strong differences for distance moved between different starvation treatments, with the exception of an inclination for non-starved insects to move farther at lower temperatures and starved insects to move farther at higher temperatures. At low temperatures, male insects appeared to move similar distances for different starvation periods, but at high temperatures, moved farther when starved. While not borne out in the analyses, these general patterns suggest that the different life stages likely differ, at least to some degree, in their response to starvation.

Reconciling our results from the laboratory and outdoor components of our study indicate that starvation clearly influences locomotor activity in terms of total distance moved, but that the effect can be variable and depend on environmental factors. In many ways, and especially for the laboratory component, our results align with many previous studies, which indicate that starvation increases searching behavior for food, and thus distance moved, in insects [13,21,28]. Orientation towards food in our study also increased when adult *B*. *hilaris* were starved compared to when they were not starved, which was consistent with previous studies [20,29]. These behavioral responses of increased dispersal and orientation towards host odors should help individuals find new sources of food. For the outdoor component of our study, however, the influence of starvation was not as straightforward, illustrating how many factors can simultaneously influence behavior of insects. The heightened response of starved individuals to temperature is a novel result and one that appears to agree with prior studies in that starvation can induce changes in behavior, although in this case, starvation is influencing dispersal by modulating how *B*. *hilaris* respond to changes in temperature. If the influence of temperature that we saw on movement is independent of light stimuli, then the changes in movement with starvation could occur with changes in temperature that occur on a daily or a seasonal basis. Previous work has also shown a strong correlation between temperature and both *B*. *hilaris* incidence on plants and fresh plant damage with greatest activity during the warmer periods of the day [22]. Activity and behavior of *B*. *hilaris* are clearly already influenced by temperature Starvation could be a critical factor of this pest’s internal state that drives responses to temperature under field conditions. For the effect of starvation on total distance moved, the pattern of greater movement for starved individuals was only present (and non-significantly) at high temperatures. Starvation can also decrease movement [13,18], and at low temperatures, starvation decreased movement in our study. These results overall do indicate that starved individuals can have different activity or foraging patterns, although responses are temperature-dependent. Our results also indicate that when the influence of starvation on behavior is being assessed, environmental factors should be addressed. While controlling these factors should be, at minimum, a component of experimental design, a better approach would be to experimentally manipulate these factors or allow them to vary.

Males displayed the greatest increases in movement with starvation in the laboratory and outdoor components of our study. In addition, starved males were more responsive to the food source in the olfactometer assay than non-starved males and females. This may be relevant to how populations of *B*. *hilaris* shift to new hosts when their hosts senesce or are destroyed. If males move farther and show and increased response to starvation, they may be the first to colonize new hosts. Male *B*. *hilaris* produce the pheromone, (E)-2-octenyl acetate, and only females are attracted to this pheromone [30,31] (De Pasquale et al. 2007; Guarino et al. 2008). While a consistent and strong attraction was not demonstrated in field settings (Palumbo et al. 2016), this pheromone still likely plays a role in dispersal dynamics. Starved males may initially colonize new food sources, with females following using both plant cues and sex pheromone to colonize plants, illustrating how effects of starvation on movement could influence populations and pest status of *B*. *hilaris*.

We found that starvation altered the turning ratio of *B*. *hilaris*, indicating that starvation can influence multiple aspects of this species’ movement. Distance moved is only one component of foraging behavior (e.g., [32]). When individuals were starved, the turning ratio was higher, which meant that movement was more directed with a greater net distance travelled for a given total distance (Table 3, Fig 3). When searching for food, insects will transition between ranging, or extensive search, when initially seeking out a patch or a resource and then intensive, or local, search within a patch or resource [21,33]. The difference in ratio that we found between non-starved and starved individuals is not a substantial enough difference that the pattern could be described as switching between intensive and extensive search behaviors. However, the increased directionality with starvation indicates that with starvation, *B*. *hilaris* move more linearly and decrease their local searching. This would increase the opportunity that an insect dispersing from a food-depleted patch finds a new patch. All stages were affected similarly and neither wind speed nor surface temperature influenced this aspect of movement. Similarly, *Pieris rapae* caterpillars tended to travel more often in a straight line, that is with higher levels of directionality, when starved [28]. While we only tested two starvation periods, we did see a pattern of greater directional movement with increasing starvation. Even longer starvation periods may have increased directionality further. A higher level of starvation could serve as a more accentuated stimuli indicating that the local patch is depleted and the insect needs to leave the patch. Conversely, lower levels of “starvation” that the insect may experience within the course of a day could increase directional movement to some degree, or it could even decrease directional movement and increase local searching. The most adaptive searching pattern in this time frame may be to search out and rely on local resources before increased levels of starvation serve as a physiological stimulus indicating movement from the patch is necessary to find food.

## Conclusions

Adults and all nymph stages of *B*. *hilaris* can cause serious damage to cruciferous crops at germinating and seedling stages. Improving our understanding of the dispersal behavior of *B*. *hilaris*, and what factors influence this behavior, is an important step towards developing an integrated pest management program for this pest. Our laboratory results show that starvation for short periods can increase distances moved through walking. Results from our outdoor experiment show that temperature is an important determinant of how starvation affects distance travelled. Starvation can affect how *B*. *hilaris* moves in search of food, with increased directionality of movement when insects are starved. Orientation of *B*. *hilaris* towards food also increased with starvation. Overall, our results improve our understanding of the movement of *B*. *hilaris* within the landscape. This is important for elucidating the role of non-crop hosts as sources of *B*. *hilaris* moving into fields and knowing what factors elicit dispersal behavior of *B*. *hilaris* between non-crop hosts or, more importantly, from non-crop hosts to nearby planted crops. A better understanding of dispersal dynamics of *B*. *hilaris* can also help develop refined management plans that account for movement of this pest using prediction of damage risk, targeted scouting, or management tactics that take advantage of movement such as trap cropping.

## Supporting information

S1 Table Replicate numbers for laboratory and outdoor experiments. Number of replicates for each stage and starvation treatment combination for analyses of total distance moved for the laboratory experiment and for analyses of total distance and turning ratio from the field experiment.
(DOCX)

S1 Fig Relationship between wind speed and air and surface temperature to account for missing wind data. To account for missing wind data, we fit a regression model to predict wind speed based on surface and air temperature measurements (*WS* = *ST* * *WS* + *ST*^2^ * *WS* + *ST*^3^ * *WS* + *AT* * *WS* + *AT*^2^ * *WS* + *AT*^3^ * *WS* + *b*), where *WS* = wind speed, *ST* = surface temperature, and *AT* = air temperature. Data for which wind speed was recorded are shaded shades of blue based on air temperature values. Points shaded red are points for which wind speed was predicted.
(TIF)
