# Supplementary material for: Influence of starvation on walking behavior of Bagrada hilaris (Hemiptera: Pentatomidae)
Source: PLoS One. 2019 Apr 18;14(4):e0215446. doi: 10.1371/journal.pone.0215446 (PMC6472788; doi:10.1371/journal.pone.0215446)
Supplement: S1 Table — Number of replicates for each stage and starvation treatment combination for analyses of total distance moved for the laboratory experiment and for analyses of total distance and turning ratio from the field experiment. (DOCX) [file pone.0215446.s001.docx]

|  |  | **Number of replicates** | | | |
| --- | --- | --- | --- | --- | --- |
|  |  | **Laboratory** |  | **Outdoors** | |
| **Stage** | **Starvation** | **Total distance** |  | **Total distance** | **Turning ratio** |
| 2nd/3rd inst. | 0 h | 26 |  | 29 | 27 |
| 4th/5th inst. | 0 h | 26 |  | 67 | 60 |
| Female | 0 h | 25 |  | 50 | 46 |
| Male | 0 h | 24 |  | 65 | 54 |
| 2nd/3rd inst. | 24 h | 26 |  | 28 | 28 |
| 4th/5th inst. | 24 h | 25 |  | 54 | 52 |
| Female | 24 h | 25 |  | 30 | 27 |
| Male | 24 h | 26 |  | 56 | 51 |
| 2nd/3rd inst. | 48 h | 25 |  | 27 | 26 |
| 4th/5th inst. | 48 h | 16 |  | 32 | 29 |
| Female | 48 h | 25 |  | 17 | 15 |
| Male | 48 h | 24 |  | 37 | 34 |
| **Total** |  | **293** |  | **492** | **449** |
