# Supplementary figures and images for: Influence of starvation on walking behavior of Bagrada hilaris (Hemiptera: Pentatomidae)
Source: PLoS One. 2019 Apr 18;14(4):e0215446. doi: 10.1371/journal.pone.0215446 (PMC6472788; doi:10.1371/journal.pone.0215446)

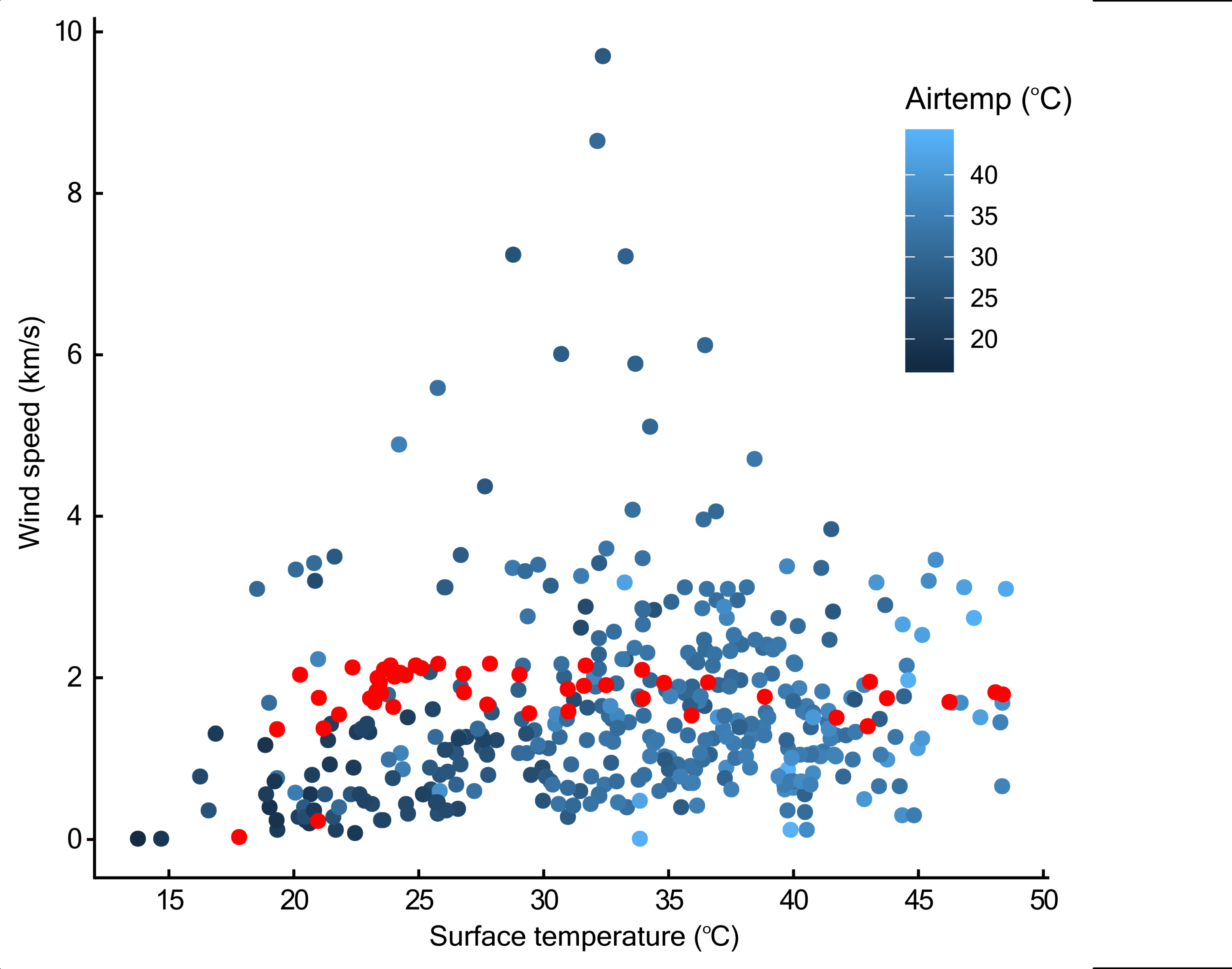

Supplement: S1 Fig — To account for missing wind data, we fit a regression model to predict wind speed based on surface and air temperature measurements (WS = ST * WS + ST2 * WS + ST3 * WS + AT * WS + AT2 * WS + AT3 * WS + b), where WS = wind speed, ST = surface temperature, and AT = air temperature. Data for which wind speed was recorded are shaded shades of blue based on air temperature values. Points shaded red are points for which wind speed was predicted. (TIF) [file pone.0215446.s002.tif]
